# Annotations

**Published:** 1907-01-12

**Authors:** 


### ANNOTATIONS.

### Congress of Climatotherapy and Urban Hygiene.

THE third Congress of Climatotherapy and Urban Hygiene will be held during the Easter vacation, 1907, on the French Riviera and in Corsica. The sessions will extend over seven days, and will be conducted at Cannes, Monaco, Mentone, and Ajaccio, but all the stations on the Mediterranean littoral are included in the programme. In 1904 the Congress met at Nice, and in 1905 at Arachon, and great efforts are being made to secure that the meeting of the present year shall rival the success of its predecessors. The local representatives of the medical profession have obtained the hearty co-operation of the municipal authorities, and adequate arrangements will be made for the convenience and enjoyment of medical visitors from foreign countries; a number of fêtes, banquets, excursions, etc., are included in the scheme of organisation. It is anticipated that a reduction of 50 per cent. in railway and steamboat charges will be secured, and that special terms for visitors will be granted by the hotels. Full information on these and other points can be obtained on application to Dr. Verdalle, 1, Boulevard d'Alsace, Cannes, who is acting as general secretary. The President of the Congress is Dr. Calmette, Member of the Academy of Medicine and Director of the Pasteur Laboratory, Lille, and a number of well-known French physicians are included among the vice-presidents. In the papers and discussions special attention will be paid to the climatic value of the French Riviera in the treatment of chronic diseases of the respiratory organs, of local tuberculosis, and of diseases of the nervous system. The Congress affords a convenient opportunity for British practitioners to make themselves familiar with the therapeutic influences to be obtained on the French Riviera.

### Boracic Fomentations.

FOUR-HOURLY boracic fomentations have for some time been the routine treatment for all superficial inflammatory or suppurative lesions, and there is probably no more efficacious or soothing application for these conditions. They have also to a large extent superseded the dressings with oil or ointment which were previously in vogue for burns and scalds. But it may be doubted whether the virtues and wide utility of this form of dressing have yet been fully realised. The qualities of a boracic fomentation are threefold. The heat increases the circulation of the part, causing a dilatation of the capillaries, and at the same time checks any hæmorrhage from the larger vessels. The boracic acid is a mild antiseptic, acting injuriously upon any germs present, but causing no injury to the tissues, and at the same time it is a mild astringent and irritant, and the moisture is soothing and prevents incrustation of any discharge and adhesion of the dressing to an open wound. A boracic fomentation, therefore, not only has the power to reduce inflammation and cleanse a wound from infection, but it also promotes healing and forms a most suitable dressing for any superficial lesion, other than a strictly aseptic surgical wound in which the edges unite by primary union. It is also a most useful form of applying moist heat to injuries of the tissues without any external wound, such as contusions, sprains, and strains. On the supposition of some power of absorption by the skin, the boracic acid may possess some virtue in these conditions and will at any rate inhibit any infective element in the skin itself. There is some skill required in the proper application of these fomentations. Different conditions require different degrees of heat and moisture, but as a general rule it is preferable to retain a certain amount of moisture in the lint, and in that case the fomentations must, of course, not be applied at boiling point. The oil silk or jaconet applied over the lint should be sufficiently large to extend beyond the margin of the lint so as to retain the moisture of the dressing.

### In Defence of Shoddy.

SOME time ago Mr. F. W. Reuss, of Dewsbury, gave a lecture to the University of Leeds Textile Society on "Shoddy, Mungo, and Rags: Their History and Importance in Woollen Manufacture." Very few people know what an important item shoddy—that substance whose name is a by-word for all that is worthless—is in English industry. Mr. Reuss estimated that every month England exports between five and six million yards of shoddy. Shoddy is made from rags, torn up and re-woven, and it is estimated that from 1,000 to 1,400 tons of wool rags are used in Yorkshire every week in the manufacture. Much of this is imported. Rags are ever an object of suspicion as possible carriers of infection, and the import of rags from places where infectious diseases are epidemic is frequently forbidden. Mr. Reuss seemed to think that these precautions are unnecessary, and declared that it had never yet been proved that a rag of any kind had conveyed infection. Some sanitary scientists have discussed the question, and have come to the conclusion that there has never been a case of disease carried from one place to another by means of rags. Mr. Reuss suggested that the existing restrictions on the importation of rags was a subject for complaint, and probably his hearers agreed with him. Yet, because shoddy is such an important manufacture —for if it did not exist the poorer classes of the community would be unable to get changes of upper clothing as frequent as for health's sake is desirable —it is important that these rags should be beyond suspicion. The confiscation of a few bags of rags is less serious, even to manufacturers, than the loss of webs of the stuff into which these rags are woven, and infinitely less serious than the risk of spreading disease. An epidemic traceable to the wearing of shoddy would damage the whole industry most severely. Mr. Reuss spoke with some acerbity of the existence of anthrax in pure wool and of the disease it causes. It is matter for regret that wool-sorters should suffer from anthrax, rare as such cases are, but we cannot see that their existence is an excuse for being more careless in bringing in rags. To add "rag-sorter's" to "wool-sorter's" disease would hardly profit anyone.

